# Effects of *Streptomyces melanosporofaciens* X216 on microbial diversity in oilseed rape soil

**DOI:** 10.3389/fpls.2024.1425798

**Published:** 2024-08-20

**Authors:** Hai-di Liang, Hu Zhou, Hui Zhao, Lin Ding, Jia Zhou, Ying-jun Zhang, Yang Gao, Zuo-hua Ren

**Affiliations:** ^1^ College of Plant Protection, Hunan Agricultural University, Changsha, China; ^2^ Yueyang Inspection and Testing Center, Yueyang, China; ^3^ Hunan Provincial Institute of Plant Protection, Changsha, China

**Keywords:** clubroot, rhizosphere soil, high-throughput sequencing, microbial community, *Streptomyces melanosporofaciens* X216

## Abstract

**Introduction:**

Clubroot disease is a devastating soil borne disease caused by infection with *Plasmodiophora brassicae*, which primarily affects cruciferous plants. The microbial diversity of the soil is an essential indicator of its quality.

**Methods:**

This study measured the physicochemical properties of the soil to study the effect of its microbial diversity on the infection of oilseed rape with *P. brassicae*. High-throughput sequences of the soil bacteria and fungi in the inter-root soils of *P. brassicae* were analyzed under different treatment conditions.

**Results:**

In the study, it was found that the efficiency of strain X216 in preventing and controlling the root disease of rapeseed was positively correlated with the amount of solution used to irrigate the root system. The results of the greenhouse and field trials showed that the efficiency of strain X216 against the root disease of rapeseed was 43.16% in the field and 62.14% in the greenhouse. Proteobacteria, Chloroflexi, Rozellomycota, and Basidiomycota are critical phylum in the development of clubroot disease. The application of biocontrol increased the relative abundance of Actinobacteria, *Bacillus, Mesorhizobium, Mycobacterium, Streptomyces* and *Filobasidium*, which affected the structure and abundance of microbial communities. A principal coordinate analysis showed that the microbial structure in the soil varied substantially in the bacterial community, and there was no significant difference in soil structure in the fungal community.

**Discussion:**

The occurrence of clubroot disease affected the structure of inter-root microbial community composition in the soil, which resulted in a decrease in its community diversity. The application of the biocontrol bacterium X216 increased the soil microbial diversity. It effectively reduced the occurrence of *P. brassicae*, and this study provides a basis to study the microbial diversity in cruciferous crops.

## 1 Introduction

Clubroot is a catastrophic soil borne disease caused by infection with *Plasmodiophora brassicae* (Siemens et al., 2009), and it significantly impacts the lifespan of plants. The latent spores of *P. brassicae.* are highly active (Hwang et al., 2015), have a high rate of infection, and are transmitted over a wide range of channels (Ahmed et al., 2011). In addition, they can survive in the soil for up to 20 years. Once infested, the soil will harbor the pathogen for an extended period, which renders it unsuitable for cruciferous plants. Which has resulted in significant production and economic losses of cruciferous vegetables in more than 60 countries worldwide. In China, cruciferous clubroot exists in the majority of provinces, and the region where it occurs is spreading, with the degree of damage rising year after year, severely limiting the development of the cruciferous crop business (Zhang et al., 2022). At present, research on clubroot at home and abroad attaches great importance, but the progress is slow, and its prevention and control technology mainly adopts measures such as screening of disease-resistant varieties, pharmaceutical control, and agronomic management, which are all less than ideal in terms of prevention and control.

Many studies have investigated that modulating the soil microbial composition between the plant roots through integrated agricultural methods has been shown to effectively control soilborne illnesses. Microbial soil treatment is an agronomic measure commonly used in current agricultural production to increase the diversity and complexity of soil-beneficial microbial populations and inhibit the propagation of pathogenic bacteria (Bhattacharyya and Jha, 2012; Wang et al., 2020). For example, tobacco green wilt (Wu et al., 2014; Liu et al., 2016), tobacco black shin disease (Kyselkova et al., 2009), cotton wilt (*Fusarium oxysporum* f. sp. *vasinfectum*) (Wang et al., 2015), and banana wilt (*F. oxysporum* f. sp. *cubense*) (Zhou et al., 2019). Recent studies demonstrated that biocontrol fungicides, not only exerted reasonable disease control but also increased the relative abundance of potentially beneficial microbial communities, such as *Bacillus amyloliquefaciens* W19 and *Mycobacterium xylosporum* NJAU4742 (Xiong et al., 2017).

In past research focused on cruciferous plants determine the relationships between soil microbial properties and plant health, and investigate key microorganisms associated with plant health in networks (Kang et al., 2024). However, the differences in physicochemical properties, microbial community composition and structure, and microbial functions of the inter-root soils of the plants through the addition of biocontrol bacteria are not known. Therefore, it is critical to explore the rhizosphere-associated bacterial and fungal communities to demonstrate the potential interaction between clubroot and microorganisms.

In the present study, a strain (X216) was isolated from the inter-root soil of healthy plants in the infected area of *P. brassicae.* and its fermented sterile filtrate showed 43-62% control of clubroot disease in oilseed rape. In this study, we hypothesized that the physicochemical properties of soil, microbial community composition, and significant enrichment of soil supplemented with biocontrol solution would be conducive to the suppression of clubroot. The objectives of this study were to (1) To study the microbial communities significantly enriched in the inter-root microorganisms of the soil. (2) the key microbial compositions associated with plant disease and disease resistance. (3) What were the effects and changes in the inter-root microbial communities of the plants after the addition of the prophylaxis solution X216?

## 2 Materials and methods

### 2.1 Experimental design and soil samples collection

The study was initiated in October 2022, and seeds of the oilseed rape variety ‘Bright Oil No. 9’ were planted in Changtang, Taiyuan Town, Hengyang County, Hunan Province, China (27°6′49′′N, 112°24′22′′E). The experiment was conducted in a randomized group design. A total of 15 rapeseed plants were planted in each hole of the field ridge (30×30 cm). There were four holes per row, two rows of one treatment, and three replicates, which were all managed in the same manner (Li et al., 2021).

To investigate the optimum treatment concentration of the X216, the treatment group was diluted 1, 2 and 5 fold, with the control group (CK) was water. The treatments were applied on the day the seeds were sown and 14 and 21 d after sowing. A volume of 500 mL of the solutions of various treatment groups per hole were rubbed over the biocontrol bacteria group in the control group (Ding et al., 2023). Plant samples were collected 45d after seedling emergence for preliminary investigation of clubroot incidence in plants.

Soil samples were collected as the crop reached maturity in 2023. The soil from oilseed rape roots was gathered by shaking the roots and removing debris, such as the large soil pieces, stones, and gravel. They were then stored in a plastic bag in a foam box. Five samples from each field were combined as a biological replicate with four replicates per treatment. The collected samples were divided into healthy (H), diseased (D), and biocontrol (DX) soil, and the samples collected from each treatment group were labeled 1-4, for a total of 12 samples. The plant roots were washed in sterile water and centrifuged. The precipitates were collected and stored at -80°C for subsequent microbial communities analysis. The diversity of microbial communities was quickly sequenced and interactively examined by the Shanghai Meiji Biomedical Science and Technology (Shanghai, China). After the soil had been naturally air-dried in the laboratory, mixed air-dried soil samples were utilized to measure the soil physicochemical parameters (Hangzhou Research Interest Information Technology Co.).

### 2.2 Methods

#### 2.2.1 Criteria used to determine the intensity of disease and quantify the effectiveness of preventative treatments

**Figure 1:**
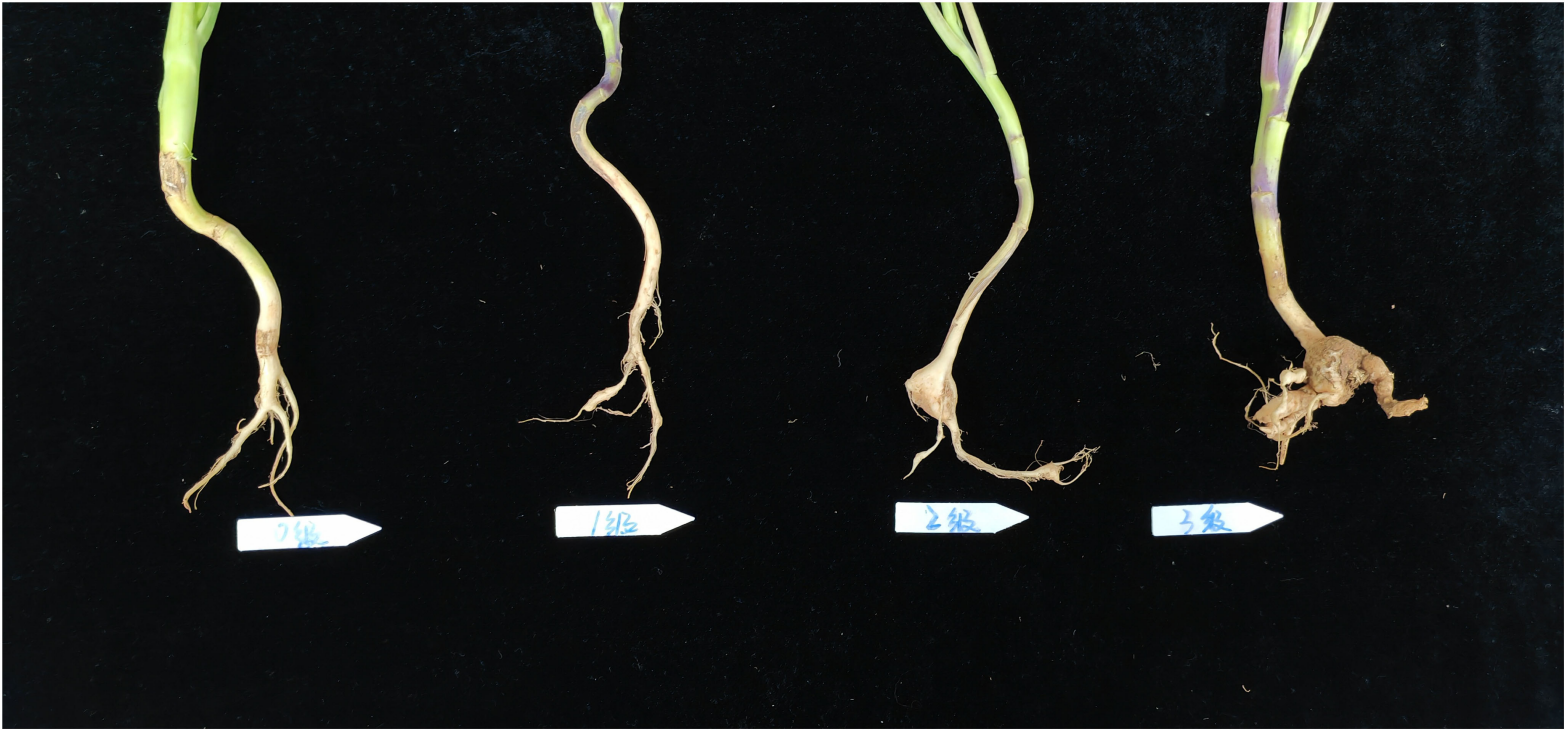
Criteria for grading clubroot disease.

The clubroot disease was classified by the criteria as follows: (NY/T, 3621-2020, Agricultural industry standard of the People’s Republic of China, 2020) (**Figure 1**): Grade 0, normal root system, asymptomatic; Grade 1, small clubbed roots < 0.5 cm in diameter formed on one-third or fewer lateral roots, no clubbing on the main root; Grade 2, one-third to two-third of the lateral roots had clubbing, or the diameter or length of clubbing on the main root < 3 times the diameter of stem base; and Grade 3, two-third or more of the lateral roots exhibited clubbing, which was obvious on the main root, and the diameter or length of clubbing > 3 times the diameter of stem base, or the clubbed roots were ulcerated.

To evaluate the optimum concentration of X216, disease incidence, disease index and control efficacy was calculated, which are shown below:


$$\begin{array}{l} \text{Disease incidence}=\text{number of diseased plants}/\\ \text{ total number of plants surveyed}\times 100\% \end{array}$$

$$\begin{array}{l} \text{Disease index}=\sum \;(\text{disease grade representative value}\\ \;\times \text{number of diseased plants at each grade})\\ \;/(\text{total number of plants}\\ \;\times \text{highest grade disease representative value})\times 100 \end{array}$$

$$\begin{array}{l} \text{Control efficiency}=[\left(\text{control disease index}-\text{treatment disease index}\right)\\ \;/\text{control disease index}]\times 100\% \end{array}$$

#### 2.2.2 Soil chemical analysis

The pH was determined by the potentiometric method. Organic matter (OM) was determined by the potassium dichromate oxidation-volumetric method. The total nitrogen (TN) level was determined using the semimicro Kjeldahl method. Alkaline dissolved nitrogen (ADN) was determined by the titrimetric method (Lu, 2000). Available phosphorus (FAP) was determined using the sodium hydrogen carbonate solution-Mo-Sb anti-spectrophotometric method (Shen et al., 2011). Fast-acting potassium (FAK) was determined by the flame photometer method. The exchangeable calcium (exchangeable Ca^2+^) was determined using the EDTA complexation-titrimetry (Bao, 2000). Exchangeable magnesium (exchangeable Mg^2+^) was determined using spectrophotometry. The cation exchange capacity (CEC) was measured by the method of Mulvaney et al. (2004).

#### 2.2.3 Molecular analyses of the soil microbial communities

To identify the diversity of the soil microbial communities, DNA was extracted from the soil samples using soil DNA Kit according to the manufacturer’s instructions. Community composition of bacteria was determined by amplifying the V3 to V4 regions of the 16S rRNA gene using 338F and 806R primers and the fungal community composition was determined by internal transcribed spacer(ITS) region using ITS1F and ITS2R primers (Zhang et al., 2023). All paired-end libraries were constructed using TruSeqTM DNA Sample Prep Kit and sequenced with Illumina MiSeq (Illumina Inc., San Diego, CA, USA).

Raw reads were trimmed and merged using FLASH (version1.2.11), filtered low-quality reads, adaptor sequences and contamination. Leveling by minimum number of sample sequences. The valid data were clustered by operational taxonomic units (OTUs) using Uparse (version11), subsequent analyses based on 97% OTUs similarity of obtained sequences (Edgar, 2013). The diversity of microbial communities were assessed by species annotation with RDP Classifier (version11.5), and taxonomy of bacteria and fungi was assigned using SILVA (version 138) and UNITE database (version 8.0), respectively. This strategy increases the accuracy of analyzing sequences (Wei et al., 2021). The alpha diversity of the bacterial and fungal communities was evaluated with Chao richness, Observed_species, Shannon index, Simpson index, Sobs index and Good_coverage using Mothur (version1.30.2). Principal coordinate analysis (PCoA) was used based on the Bray–Curtis dissimilarity matrix to reflect beta diversity (Xi et al., 2023), with an One-way ANOVA bar plot analysis employed to determine whether the differences among groups were significant. And the function of bacteria community and fungal community was predicted using PICRUSt2 (version 2.2.0).

#### 2.2.4 Statistical analysis

The descriptive data of field survey, physicochemical properties, and alpha diversity were represented as the mean ± standard error. To examined data normality and homogeneity of variance test, the Shapiro –Wilk test and Levene’s test were performed using SPSS 26.0 (IBM, Inc, Armonk, NY, USA). The soil physicochemical and alpha-diversity indices among H, D, DX groups were analyzed using a one-way ANOVA followed by an Duncan’s new multiple range test using SPSS (the normal distribution was verified as well as whether the variance satisfied chi-square before data analysis) (*P* < 0.05).

## 3 Results

### 3.1 Efficacy of X216 at controlling clubroot infection in an oilseed rape field

**Figure 2:**
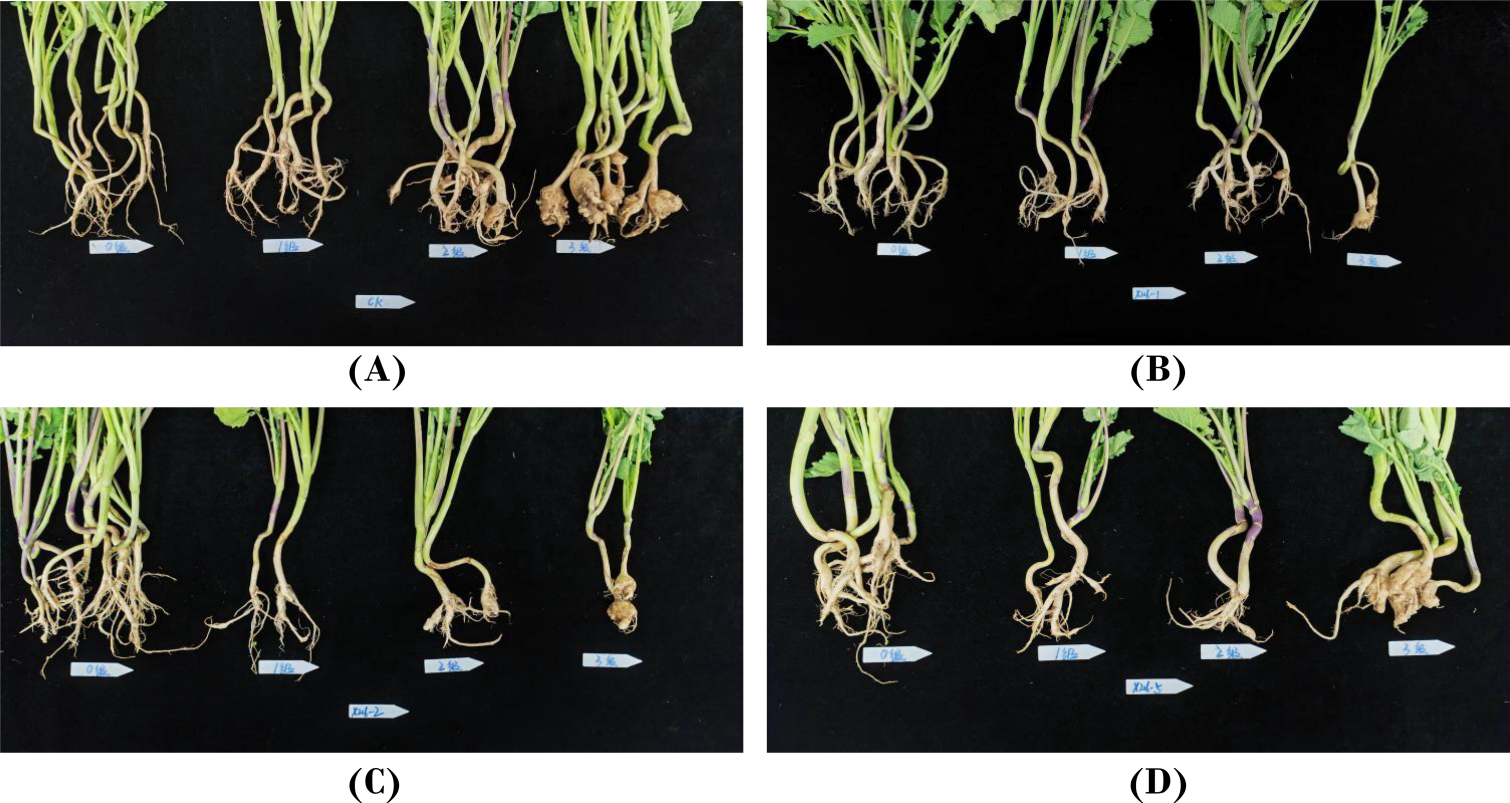
Field control of *Plasmodiophora brassicae* in oilseed rape with *Streptomyces melanosporofaciens* X216. **(A)**: CK; **(B)**: *S. melanosporofaciens* X216 culture stock solution; **(C)**: *S. melanosporofaciens* X216 culture diluted 2-fold; **(D)**: *S. melanosporofaciens* X216 culture diluted 5-fold.

The field survey grading schematic is shown in **Figure 1**, according to the survey grading standards for the treatment group X216 and the control group is graded in **Figure 2**. The control effect of biocontrol solution X216 on clubroot diminished with the increase of dilution times, and the control effect of the original solution of biocontrol bacteria on clubroot was the best, which could reach 54.21% (**Table 1**). The field testing revealed that the use of the X216 bacterial solution in oilseed rape field trials substantially reduce the disease index for clubroot infection in this crop.

**Table 1:** Field control of *Plasmodiophora brassicae* in oilseed rape with *Streptomyces melanosporofaciens* X216.

Treatment	Dilution ratio	Disease incidence/%	Disease index	Control efficiency/%
CK	–	76.77 ± 1.78^a^	60.50 ± 1.27^a^	–
X216	1	45.27 ± 3.57^c^	27.70 ± 2.34^c^	54.21 ± 3.87^a^
	2	51.28 ± 8.79^c^	32.573 ± 4.47^c^	46.15 ± 7.39^a^
	5	64.69 ± 3.19^b^	41.29 ± 2.64^b^	31.75 ± 4.36^b^

Data are mean ± SE. Different letters in the same column indicate significant difference at the *P* < 0.05 level.

### 3.2 Physicochemical characterization of the inter-root soil

No significant association was observed for pH, TN, ADN, FAP, OM exchangeable Ca^2+^ and CEC with the presence of the soils of healthy (H), diseased (D) and biocontrol (DX) soil (*P*>0.05) (**Table 2**). Compared with the healthy soil, the FAK content was significantly higher in biocontrol soil (*P*< 0.05).Compared with the diseased soil, the exchangeable Mg^2+^ content was significantly higher in biocontrol soil (*P*< 0.05).

**Table 2:** Determination of physico-chemical properties of inter-root soil of oilseed rape plants under different treatment conditions.

	H(*n*=3)	D(*n*=3)	DX(*n*=3)
pH	6.15 ± 0.52	5.84 ± 0.09	6.44 ± 0.30
TN (g·kg^−1^)	1.62 ± 0.26	1.76 ± 0.18	1.83 ± 0.26
ADN (g·kg^−1)^	127.78 ± 14.38	143.59 ± 23.74	139.98 ± 18.43
FAP (g·kg^−1^)	23.61 ± 8.85	22.43 ± 3.45	19.20 ± 5.03
FAK (g·kg^−1^)	111.41 ± 48.13^b^	322.81 ± 39.11^ab^	382.30 ± 186.21^a^
OM (g·kg^−1^)	26.15 ± 1.73	32.30 ± 3.12	33.92 ± 5.63
exchangeable Ca^2+^ (g·kg^−1)^	12.20 ± 0.94	12.69 ± 0.33	12.30 ± 1.07
exchangeable Mg^2+^ (g·kg^−1^)	3.38 ± 0.25^ab^	2.50 ± 0.25^b^	4.40 ± 1.17^a^
CEC(g·kg^−1)^	12.84 ± 1.96	13.59 ± 2.76	12.46 ± 1.29

Data are mean ± SE. Different letters in the same row indicate significant difference at the P < 0.05 level, the same below.

H, healthy soil; D, diseased soil; DX, biocontrol soil. TN, total nitrogen; AND, alkaline dissolved nitrogen; FAP, effective phosphorus; FAK, available potassium; OM, organic matter; exchangeable Ca^2+^; exchangeable calcium; exchangeable Mg^2+^, exchangeable magnesium; CEC, cation exchange capacity.

### 3.3 Cluster analysis of the inter-root soil OTUs

Sequencing results of 12 soil samples from Hunan province were summarized (**Table 3**). The bacterial dilution curve of the soil sample tended to flatten when sequenced at a depth of 3,500 X (**Figure 2A**). The fungal dilution curve of the soil sample tended to flatten at sequencing depths of 5,000X (**Figure 2B**). The characteristics of changes in the soil microbial community in the inter-root soil of oilseed rape can be more accurately reflected by the reasonableness of the sample data.

**Table 3:** Statistics of the sequencing analysis of the soil microbes.

	Bacteria			Fungi		
Samples name	Sequence	Number of bases/bp	Average length/bp	Sequence	Number of bases/bp	Average length/bp
H1	91584	37935653	414.22	67378	16525486	245.27
H2	58360	24173316	414.21	67747	16498955	243.54
H3	95304	39556999	415.06	75159	18361679	244.30
H4	82696	34250541	414.17	83540	21576625	258.28
D1	84644	35350300	417.64	90822	21266103	234.15
D2	97807	40686316	415.99	92736	22223563	239.64
D3	91772	38333301	417.70	77137	21445746	278.02
D4	92948	38535008	414.59	75328	18472372	245.23
DX1	90802	37663838	414.79	69401	16979526	244.66
DX2	97295	40336407	414.58	54403	13534057	248.77
DX3	86551	36144399	417.61	62870	16110879	256.26
DX4	78868	32610893	413.49	60447	15265139	252.54

**Figure 3:**
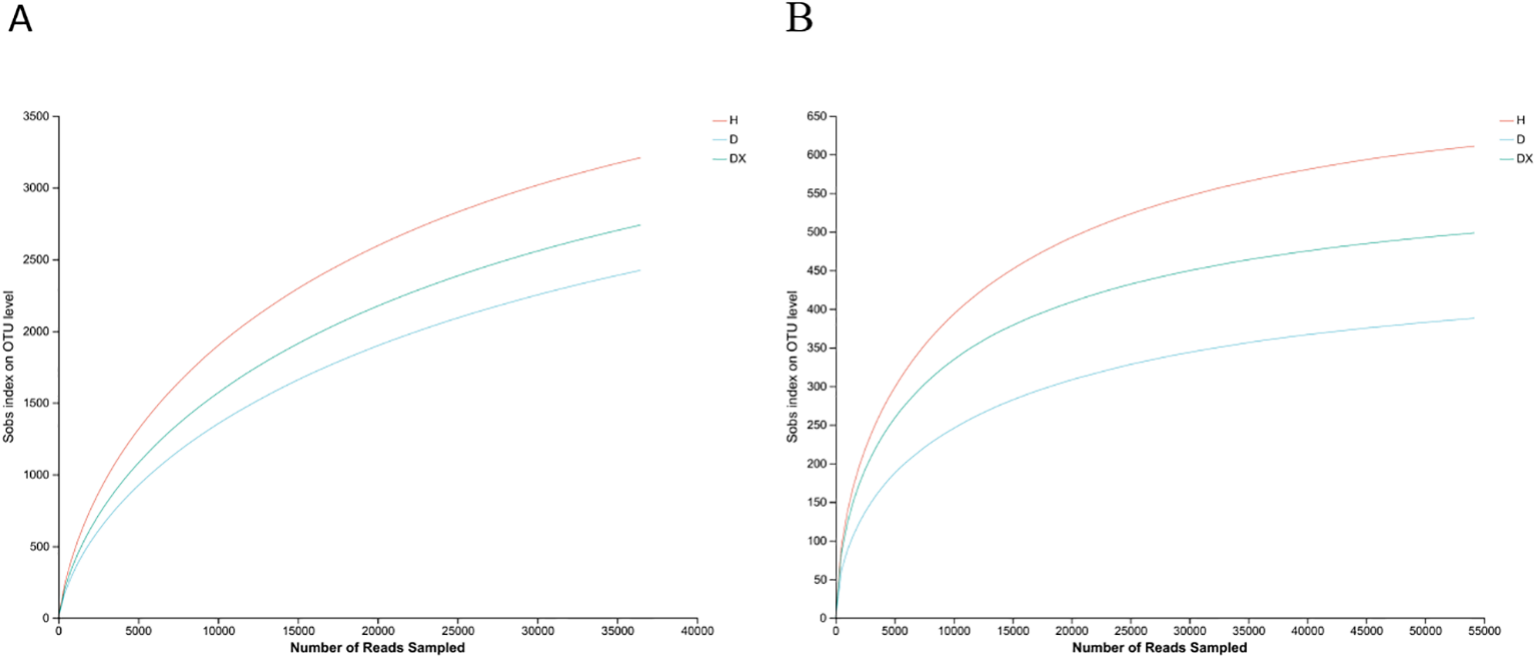
Inter-radial soil thinning curves in oilseed rape plants. **(A)** Bacteria; **(B)** Fungi.

**Figure 4:**
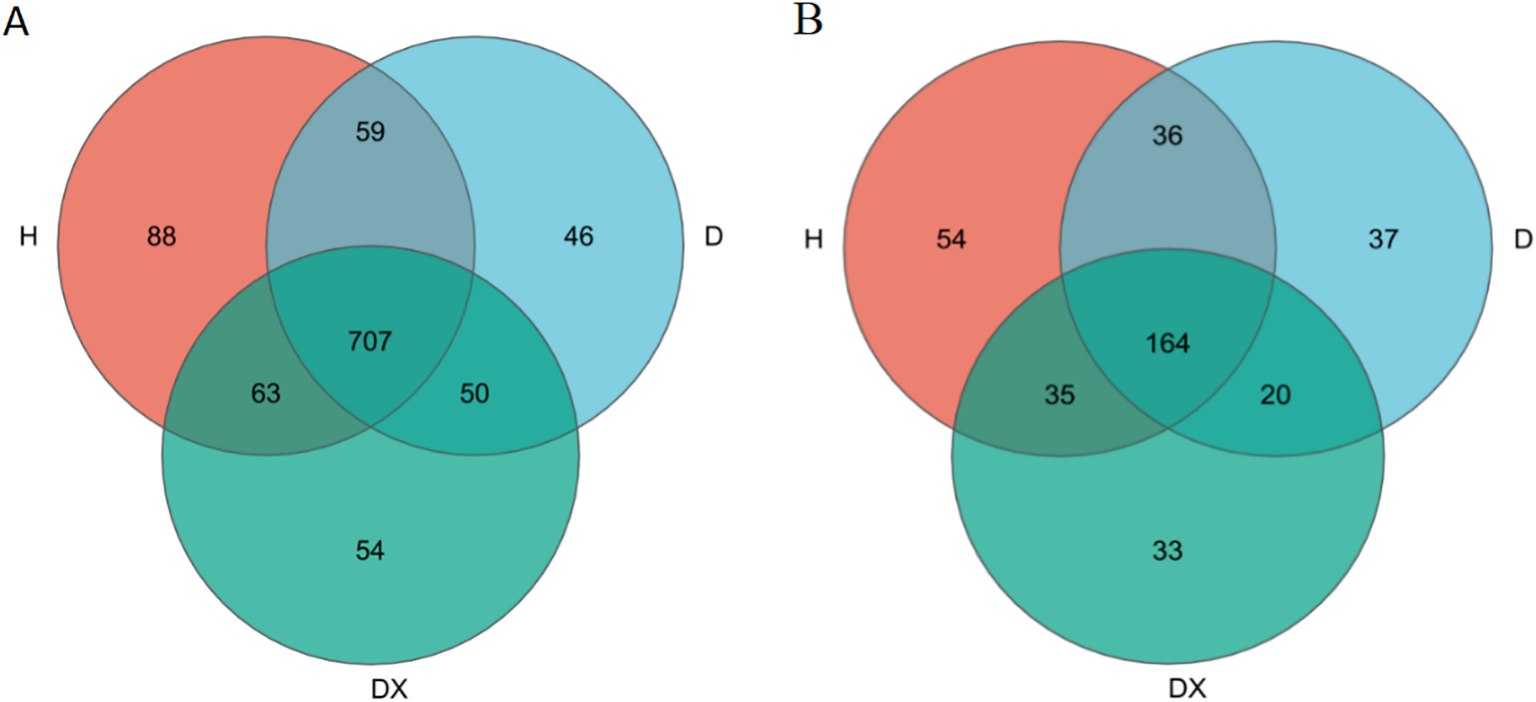
Venn diagram of the microbial number in the oilseed rape plants’ inter-root soil. **(A)** Bacteria; **(B)** Fungi.

According to the results of a cluster analysis of the OTUs from the inter-root soil, there were 707 bacterial genus in total shared by the inter-root soils of healthy (H), diseased (D) and biocontrol (DX) (**Figure 3**). A total of 164 fungal genus were shared by the inter-root soils of H, D, and DX (**Figure 4**). The number of OTUs in the inter-root soil microbial community of biocontrol oilseed rape plants was higher than that of diseased plants, and the number of OTUs in the inter-root soil microbial community of diseased oilseed rape plants was lower than that of healthy plants.

### 3.4 Microbial alpha and beta diversity analysis in the inter-root soils

The results of the alpha-diversity study are be shown in **Table 4**. The fungal community coverage exceeded 0.99, and the bacterial community exceeded 0.97. In the bacterial community, compared with diseased soil microbial community, Shannon index and sobs index of healthy soil was higher and significantly different (*P*<0.05). Compared with healthy soil microbial community, Ace, Chao 1, Shannon and sobs index of biocontrol soil was lower and not significantly different (*P*>0.05). In the fungal community, compared with healthy soil microbial community, Ace, Chao 1 and sobs index of diseased soil was lower and significantly different (*P*<0.05), and the difference was not significant in the biocontrol soil.

**Table 4:** Alpha-diversity index table.

Samples		Ace index	Chao 1 index	Shannon index	Simpson index	Coverage index	Sobs index
Bacteria	H	4084.93 ± 378.29	3873.52 ± 261.24	6.16 ± 0.25^a^	0.015 ± 0.052	0.974 ± 0.004^a^	3207.75 ± 123.60^a^
	D	3396.83 ± 378.29	3207.08 ± 363.72	5.19 ± 0.53^b^	0.037 ± 0.023	0.976 ± 0.002^a^	2323.00 ± 360.73^b^
	DX	3671.84 ± 440.71	3493.03 ± 373.07	5.75 ± 0.32^ab^	0.018 ± 0.080	0.975 ± 0.003^a^	2738.50 ± 322.93^b^
fungi	H	660.60 ± 85.85^a^	654.91 ± 94.57^a^	3.51 ± 0.63	0.103 ± 0.078	0.998 ± 0.000	610.75 ± 91.13^a^
	D	429.82 ± 139.98^b^	427.41 ± 140.78^b^	2.73 ± 0.69	0.201 ± 0.089	0.998 ± 0.000	388.00 ± 140.13^b^
	DX	541.81 ± 73.63^ab^	539.3 ± 71.26^ab^	3.37 ± 0.24	0.103 ± 0.039	0.998 ± 0.001	498.50 ± 54.77^ab^

Data are mean ± SE. Different letters in the same column indicate significant difference at the *P* < 0.05 level.

**Figure 5:**
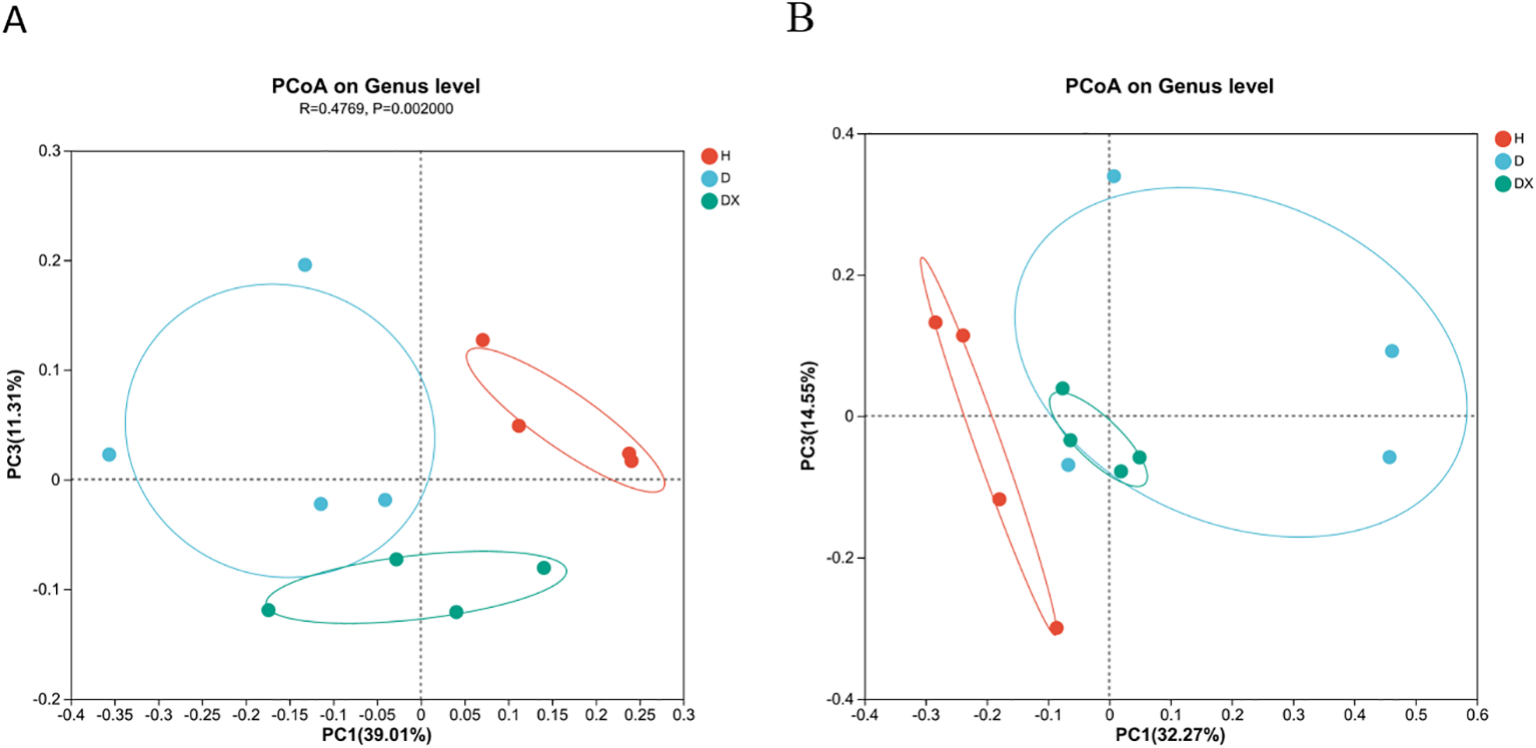
Principal coordinate analyses (PCoA) of the inter-root soil of oilseed rape plants. **(A)** Bacteria; **(B)** fungi.

Overall similarities in bacterial community structures among samples were displayed using principal component analysis (PCoA) (**Figure 5**). Principal Coordinate Analysis (PCoA) based on Bray-Curtis calculated distances, the contribution of the bacterial community in the first and third principal coordinates was 39.01% and 11.31% respectively; that of the fungal community was 32.27% and 14.55% respectively. In the bacterial community, healthy soil was significantly segregated compared to diseased and biocontrol soil at the genus level (*P* < 0.05). There were significant differences in the community composition of healthy, diseased and biocontrol soil. In the fungal community, there was no significant separation of the healthy, diseased and biocontrol soil, diseased and biocontrol soil community composition at the genus level. Healthy had obvious separation tendency from diseased and biocontrol soil respectively, and the microbial community composition of healthy is different. However, diseased and biocontrol soil showed no obvious separation trend, and the community composition was similar.

### 3.5 Community composition analysis of the inter-root soils

#### 3.5.1 Bacterial composition

**Figure 6:**
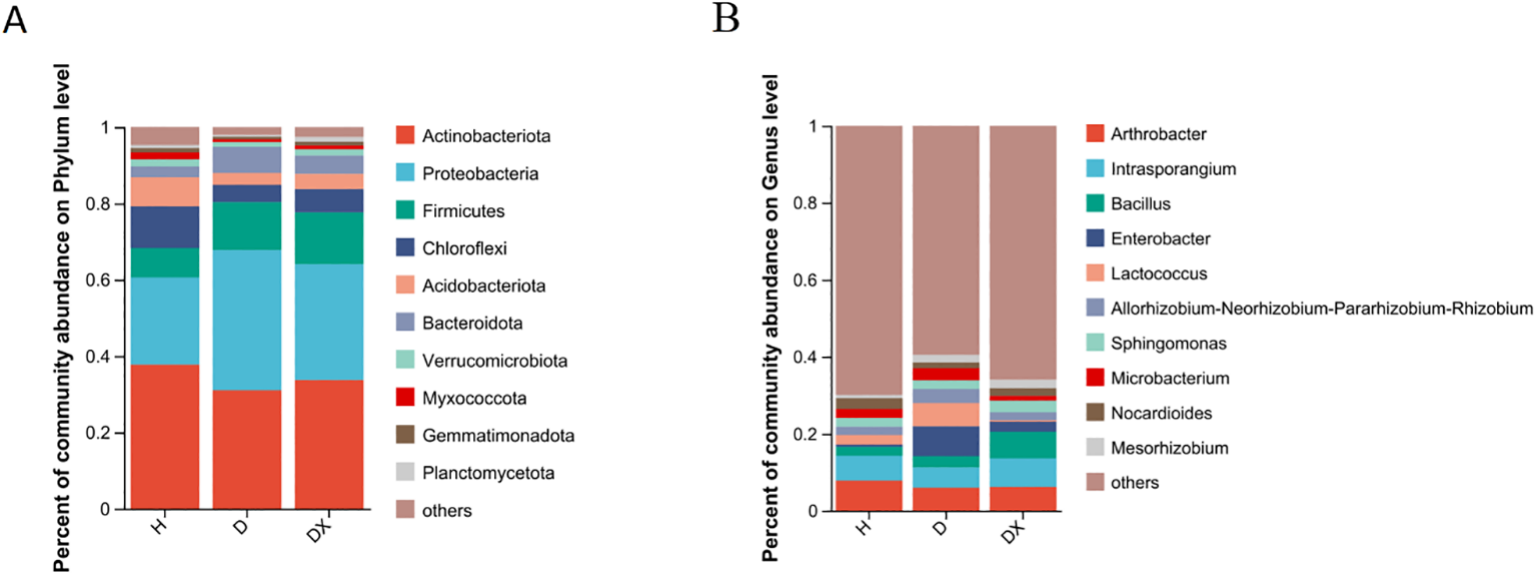
Community composition of microorganisms in bacterial communities. **(A)** Distribution of community composition at the phylum level. Others represent genus of low relative abundance that rank lower than 10. **(B)** Distribution of community composition at the genus level. Others represent genus of low relative abundance that rank lower than 10.

**Figure 7:**
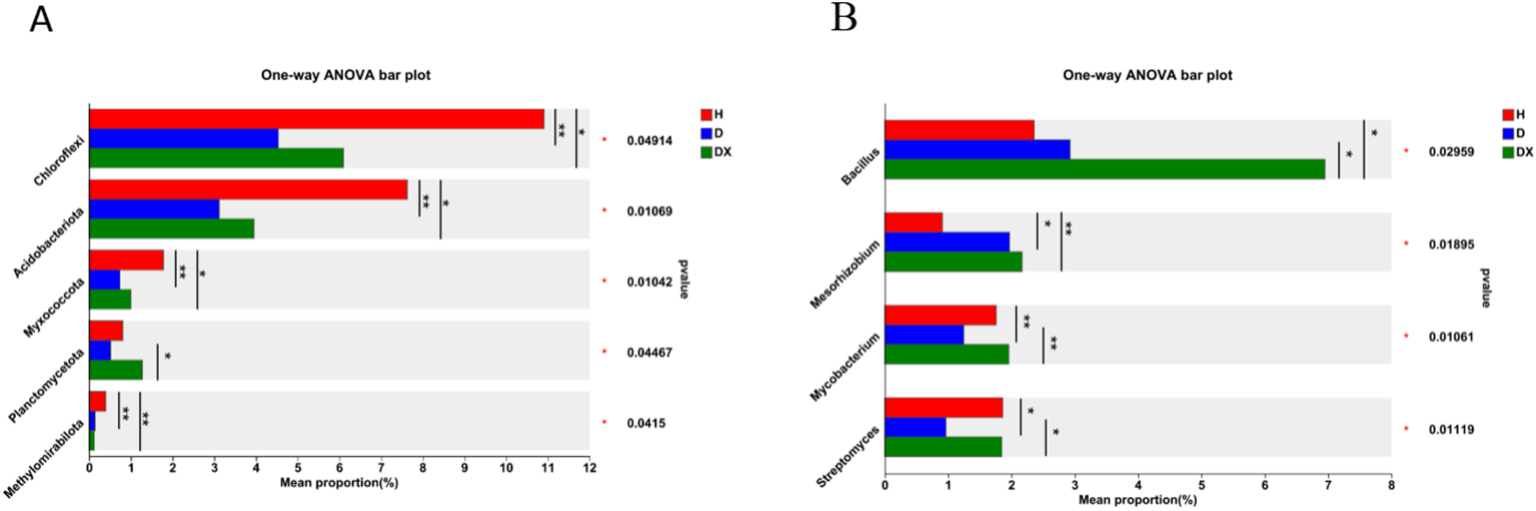
Species analysis of bacterial communities with significant differences at different levels. **(A)** The relative abundance of H, D and DX species is significantly different at the phylum level. **(B)** The relative abundance of H, D and DX species was significantly different at the genus level. (*P < 0.05; **P < 0.01;** *P* < 0.001).

Actinobacteria, Proteobacteria, Firmicutes, Chloroflexi, Bacteroidota, and Acidobacteria were the dominant phylum in the bacterial community. The composition of the main dominant phylum of inter-root soil bacteria was consistent in healthy, diseased and biocontrol soil, but the abundance of each differed significantly (**Figure 6A**). The relative abundance of Chloroflexi, Acidobacteriota, Myxococcota, Planctomycetota, and Methylomirabilota was significantly different under the comparison of the healthy, diseased and biocontrol soil (*P* < 0.05) (**Figure 7A**). The relative abundance of Chloroflexi, Acidobacteria, Myxcococcota and Methylomirabilota were highly significantly lower in diseased soil than healthy soil (*P*<0.01), and also considerably lower in biocontrol soil (*P*<0.05). The relative abundance of Planctomycete was significantly higher in the biocontrol soil than in the diseased soil (*P*<0.05), while not significantly different from the healthy soil. *Arthrobacter*, *Intrasporangium*, *Bacillus*, *Enterobacter*, and *Lactococcus* were the dominant genus in the bacterial community (**Figure 6B**). And *Lactococcus* and *Enterobacter* had the highest relative abundance in group diseased soil; *Bacillus* had the highest relative abundance in group biocontrol soil. Under the comparison of genus level, *Bacillus*, *Mesorhizobium*, *Mycobacterium*, and *Streptomyces* were significantly different in healthy, diseased, and biocontrol soil (*P* < 0.05) **Figure 7B**. Biocontrol soil had significantly higher levels of bacillus than the healthy and diseased soil (*P*<0.05). A more in-depth analysis of the differences in the relative abundance of microbial composition showed that the relative abundance of *Mycobacterium*, *Streptomyces*, were significantly higher in group biocontrol soil than in diseased soil (*P*<0.05), whereas it was similar to healthy soil. The relative abundance of *Mesorhizobium* was significantly higher in the diseased soil than in the healthy soil (*P* < 0.05).

#### 3.5.2 Fungal composition

**Figure 8:**
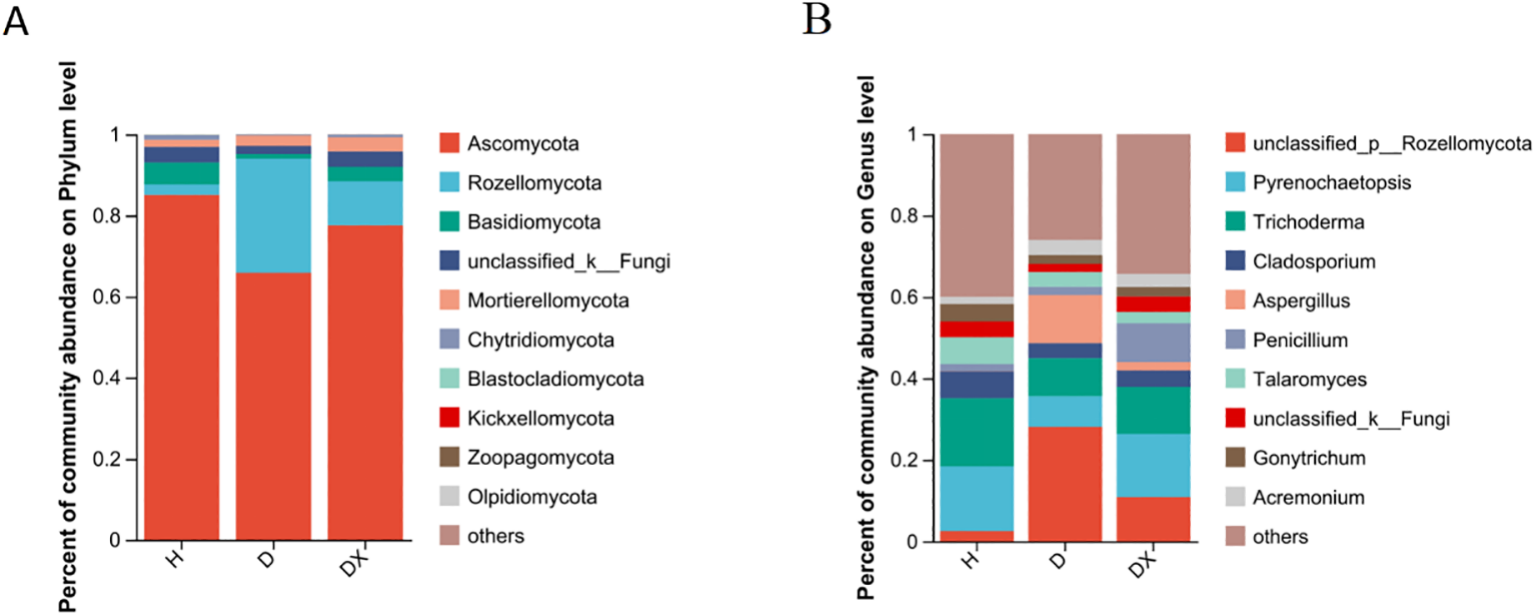
Community composition of microorganisms in Fungi communities. **(A)** Distribution of community composition at the phylum level.Others represent genus of low relative abundance that rank lower than 10. **(B)** Distribution of community composition at the genus level. Others represent genus of low relative abundance that rank lower than 10.

**Figure 9:**
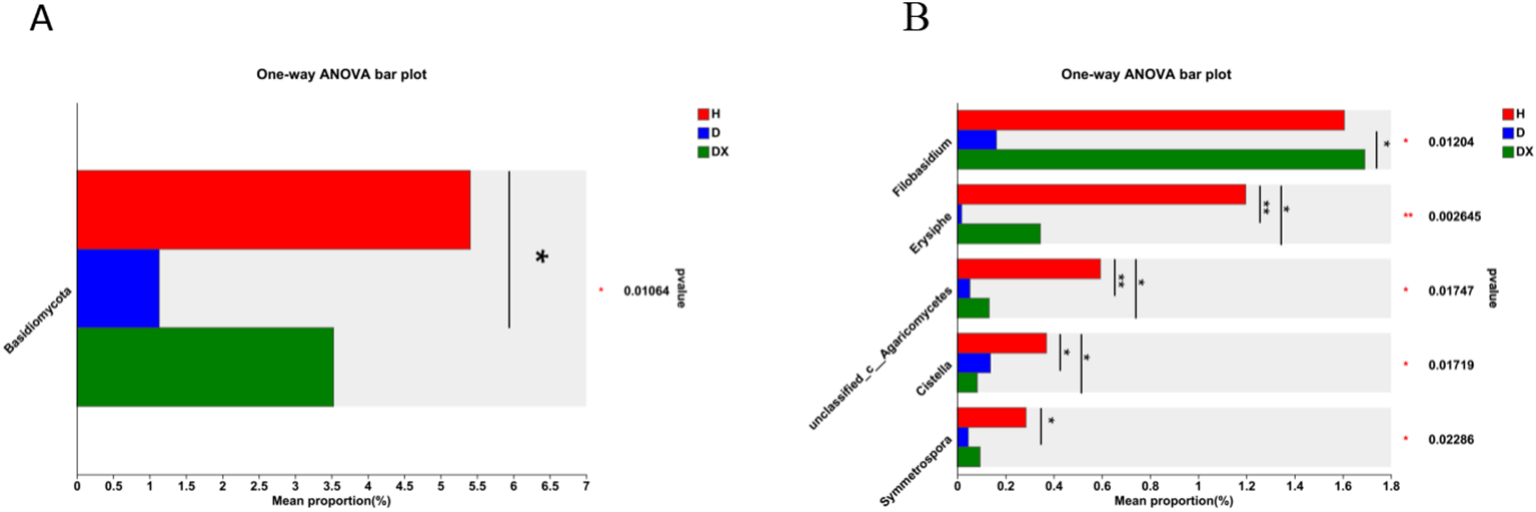
Species analysis of fungi communities with significant differences at different levels. **(A)** The relative abundance of H, D and DX species is significantly different at the phylum level. **(B)** The relative abundance of H, D and DX species was significantly different at the genus level. (*P < 0.05; **P < 0.01; **P < 0.001).

Ascomycota, Rozellomycota, Basidiomycota and Mortierellomycota are the four dominant fungal phylum in the fungal community(**Figure 8A**). Ascomycota was the most significant dominant fungal phylum in soil with a relative abundance of 85.08% in healthy soil. Rozellomycota was the second most dominant phylum with a relative abundance of 28.12% in diseased soil (**Figure 8A**). Basidiomycota differed significantly in healthy, diseased, and biocontrol soil (*P* < 0.05) (**Figure 9A**). *Unclassified:p:Rozellomycota*, *Pyrenochaetopsis*, and *Trichoderma* were the three dominant fungal genera (**Figure 8B**). *Filobasidium*, *Erysiphe*, *unclassified_c:Agaricomycetes*, *Cistella*, and *Symmetrospora* were significantly different in healthy, diseased and biocontrol soil (*P* < 0.05) (**Figure 9B**). The relative abundance of *Erysiph, unclassified_c:Agaricomycetes* and *Cistella* were lower in diseased compared to healthy soil (*P* < 0.05), but biocontrol soil difference is not significant.

### 3.6 Community prediction of microbial function in the inter-root soil of oilseed rape

**Figure 10:**
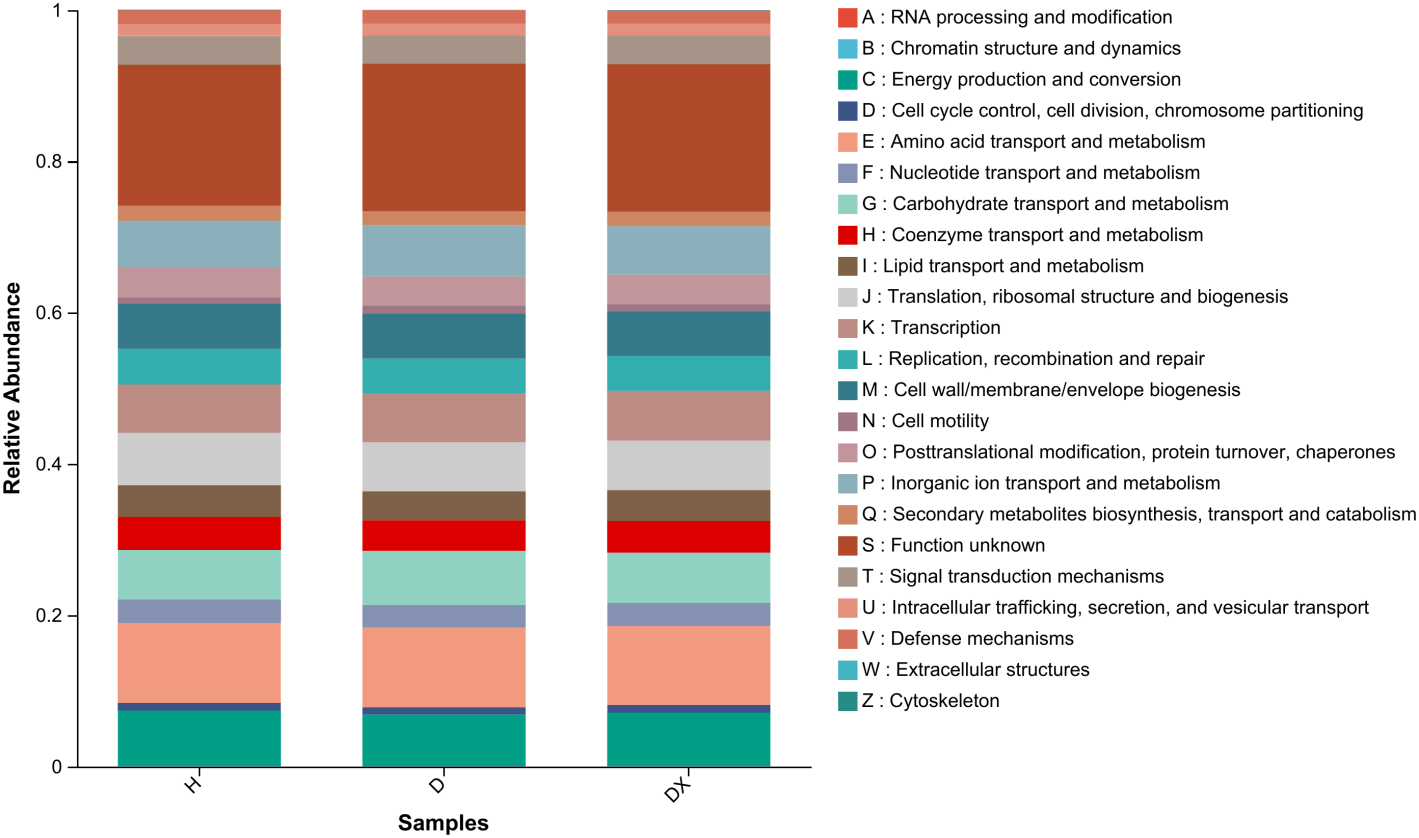
Functional prediction of the soil bacterial community in the root perimeter of oil seed rape plants.

To investigate the differences in the functional distribution of inter-root soil microbial communities among the healthy, diseased and biocontrol soil, the bacterial communities were functionally predicted using PICRUSt2 (**Figure 10**). Metabolism, Genetic Information Processing, Environmental Information Processing, Cellular Processes, Human Diseases, and Organic Systems were the six types of bacterial functions identified in the inter-root soil bacterial community of oilseed rape plants.

**Figure 11:**
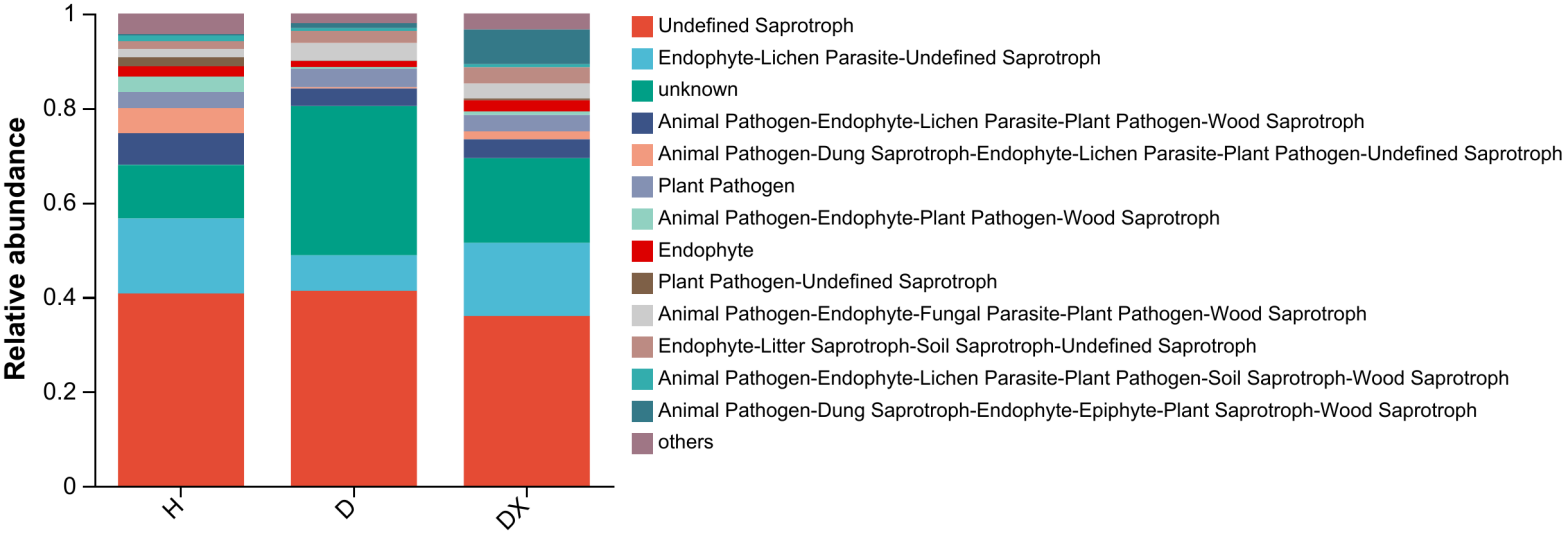
Functional prediction of the soil fungal community in the root perimeter of oil seed rape plants.

The functional classifications of fungi in the soil samples and the abundance information of each functional classification in the different samples was classified and analyzed by a FUNGuild functional prediction (**Figure 11**). This prediction showed that the inter-root soils of healthy, diseased, and biocontrol soil had a relatively high abundance of three taxa of fungal, namely Undefined Saprotroph, Endophyte-Lichen Parasite-Undefined Saprotroph and Endophyte-Lichen Parasite-unknown. Undefined Parasite-unknown had higher relative abundances in the inter-root soil of healthy plants. The functional abundance of Undefined Saprotroph was 40.74%, 41.30%, and 35.94% in healthy, diseased, and biocontrol soil of plants, respectively.

## 4 Discussion

Physical and chemical attributes of soil can operate in the suppression of plant diseases directly or indirectly through their impact on the activity of soil microorganisms (Mazzola, 2002). The results of soil physical and chemical properties in this study showed that the inter-root soils of the diseased plants had higher levels of TN, ADK, OM, exchangeable Ca^2+^, and CEC than the soils of healthy plants. The diseased plants had higher levels of ADN, FAP, and CEC, but a lower pH and lower levels of TN, FAK, OM and exchangeable magnesium than those with biocontrol soil. Biocontrol soil significantly increased the content of FAK and exchangeable magnesium. Yan et al. (2018) analyzed the N, P, and K contents of rhizomatous mustard soil, and found that the N and K contents were higher in rhizomatous soil, and the disease was aggravated with the increase of N and P contents. Similar results were obtained in this study.

The analysis of alpha-diversity in this study indicated that the richness of soil microorganisms in the inter-root of healthy plants was higher than that of diseased plants, and the number of species was more evenly distributed. *Plasmodiophora brassicae* infestation leads to reduced diversity of soil bacterial communities, which resulted in changes in the soil microbiology. Biocontrol soil increased the abundance and diversity of microorganisms to some extent. The introduction of soilborne pathogens results in alterations to the composition and organization of the inter-root microbial community (Erlacher et al., 2014). The study showed that a well microbial community structure improves the ability of plants to absorb nutrients from the soil (Bulgarelli et al., 2013), increases their resilience to abiotic stresses, and shields the host plants from deleterious organisms (Berendsen et al., 2012; Liu et al., 2020). That indicated the application of a biocontrol solution increased the diversity and abundance of the bacterial and fungal communities, while reducing the incidence of clubroot and providing good control of this pathogen.

The primary dominant phylum of the inter-root soil bacteria remained stable across the treatment settings. Differences in the structure of diseased and healthy soil bacterial communities were contributing factor in the development of clubroot in oilseed rape. A comparison of the populations of soil inter-root microorganisms that differed significantly between healthy and diseased plants can enable searches for effective microbial populations related to soil disease suppression or susceptibility to disease (Liu et al., 2018). It has been found that the relative abundance of Proteobacteria, Rozellomycota, *Mesorhizobium*, and *Aspergillus* in diseased soil is relatively high, which may be related to the incidence of root disease. And the application of biocontrol increased the relative abundance of Actinobacteria, *Bacillus*, and *Mycobacterium*, which affected the structure and abundance of microbial communities, it may play an important role in the prevention and treatment of root swelling disease. Consistent with the findings of Ambrosini et al. (2016) and Li and Zhao (2017) who showed that the application of microbial fungicides can increase the number of bacteria and fungal in the soil. Many studies have demonstrated that a diverse microbial community is often less prone to pathogen invasion than a simpler microbial community (Fargione and Tilman, 2005; Van Elsas et al., 2012; Bonanomi et al., 2014; Chen et al., 2019). At present, *Bacillus* subtilis, and *Trichoderma* harzianum have been well-used in a variety of crops.

The PICRUSt2 analysis showed that the inter-root soil bacterial communities of healthy, diseased, and biotrophic X216 plants were characterized by four major functional groups, including Metabolism, Environmental information processing, Cellular processes, and Organic systems, with a higher abundance of metabolism (Zhang et al., 2023). A higher abundance of beneficial bacteria, such as *Intrasporangium*, *Bacillus*, and *Enterobacter*, and a higher abundance of the fungal *Pyrenochaetopsis*, *Trichoderma*, *Talaromyces*, and *Cladosporium* were detected in the inter-root soil of healthy oilseed rape. This study also identified some unknown bacteria and fungal that were only present in the inter-root soil samples of healthy oilseed rape plants, and these microbial may be potential biocontrol resources for the control of clubroot. In addition, a variety of pathogenic bacteria from other crops were also detected in the inter-root soil of healthy oilseed rape plants, and the effects of these pathogenic bacteria on non-host plants, such as oilseed rape, need to be further investigated. The use of FUNGuild to predict the functionality of fungal communities showed that there was a high functional abundance of the two groups that were members of saprotrophic and phytopathogenic groups, and some typical saprotrophic fungal or pathogenic bacteria were detected in the inter-root soil samples of the oilseed rape plants, such as *Fusarium*, *Erysiphe*. This suggests that the occurrence of clubroot affects the composition of the microbial community, which may lead to the alteration of its function and ultimately cause an imbalance in the community of soil microbial.

## 5 Conclusion

The effect of biological control solution X216 on root swelling decreased with the increase of dilution ratio, and the best control effect reached 54.21%. The physical and chemical properties of D, H, DX soil and microbial community composition were analyzed that biocontrol has great influence on exchangeable magnesium. It was studied that the application of biocontrol increased the relative abundance of Planctomycetota, *Bacillus*, *Mesorhizobium*, *Mycobacterium*, *Streptomyces* and *Filobasidium*, which affected the structure and abundance of microbial communities. Significant differences were found between the healthy and diseased soils in Chloroflexi and Acidobacteriota, Myxococcota, and the application of biocontrol significantly increased the relative abundances of Planctomycetota. The FUNGuild to predict the functionality of fungal communities showed that there was a high functional abundance of the two groups that were members of saprotrophic and phytopathogenic groups. This study also identified some unknown bacteria and fungal that were only present in the inter-root soil samples of healthy oilseed rape plants, and these microbial may be potential biocontrol resources for the control of clubroot. In addition, a variety of pathogenic bacteria from other crops were also detected in the inter-root soil of healthy oilseed rape plants, and the effects of these pathogenic bacteria on non-host plants, such as oilseed rape, need to be further investigated. Therefore, the sustainable and effective green prevention and control of root and tuber diseases in cruciferous crops can be realized by regulating the structure of soil microbial community, and this type of research merits a more in-depth understanding of the microbial community related to clubroot in conjunction with biocontrol.

## Data Availability

The original contributions presented in the study are publicly available. This data can be found at the National Center for Biotechnology Information (NCBI) using accession number PRJNA1108504.
